# Nothing as Practical as a Good Theory? The Theoretical Basis of HIV Prevention Interventions for Young People in Sub-Saharan Africa: A Systematic Review

**DOI:** 10.1155/2012/345327

**Published:** 2012-08-01

**Authors:** Kristien Michielsen, Matthew Chersich, Marleen Temmerman, Tessa Dooms, Ronan Van Rossem

**Affiliations:** ^1^International Centre for Reproductive Health, Faculty of Medicine and Health Sciences, Ghent University, De Pintelaan 185 P3, 9000 Ghent, Belgium; ^2^Centre for Health Policy, School of Public Health, University of the Witwatersrand, Johannesburg 2000, South Africa; ^3^Department of Sociology, Faculty of Political and Social Sciences, Ghent University, 9000 Ghent, Belgium

## Abstract

This paper assesses the extent to which HIV prevention interventions for young people in sub-Saharan Africa are grounded in theory and if theory-based interventions are more effective. Three databases were searched for evaluation studies of HIV prevention interventions for youth. Additional articles were identified on websites of international organisations and through searching references. 34 interventions were included; 25 mentioned the use of theory. Social Cognitive Theory was most prominent (*n* = 13), followed by Health Belief Model (*n* = 7), and Theory of Reasoned Action/Planned Behaviour (*n* = 6). These cognitive behavioural theories assume that cognitions drive sexual behaviour. Reporting on choice and use of theory was low. Only three articles provided information about why a particular theory was selected. Interventions used theory to inform content (*n* = 13), for evaluation purposes (*n* = 4) or both (*n* = 7). No patterns of differential effectiveness could be detected between studies using and not using theory, or according to whether a theory informed content, and/or evaluation. We discuss characteristics of the theories that might account for the limited effectiveness observed, including overreliance on cognitions that likely vary according to type of sexual behaviour and other personal factors, inadequately address interpersonal factors, and failure to account for contextual factors.

## 1. Introduction

With an estimated 2.7 million new infections worldwide in 2010, HIV incidence remains at very high levels [1]. Sub-Saharan Africa, accounting for 70% of these infections, remains particularly affected. About 40% of new HIV infections occur in the age group 15 to 24 years [1]. Therefore, targeted prevention programmes for young people are essential in reversing the HIV epidemic [2, 3]. Over the past decades, a considerable number of HIV prevention interventions for young people in sub-Saharan Africa have been developed, implemented, and evaluated. Nevertheless, even though these interventions seem to increase knowledge and encourage positive attitudes, radical changes in sexual behaviour have not occurred [4, 5].

Theory is said to be an essential component of successful health promotion interventions [6, 7]. Behavioural theory can assist to understand the determinants of risky and safe sexual behaviour [8] and hence help to identify underlying principles about how people change their behaviour [9]. Further, it aims to explains why and how behaviours occur and allows us to predict future behaviours by establishing relationships between key variables. Beyond providing constructs, processes and hypotheses for setting up interventions, theories can also provide the basis for testing the effectiveness of interventions [10]. Furthermore, theories can serve as a framework for accumulating knowledge [11]. Reviews that assessed the theoretical underpinnings of behavioural interventions for young people worldwide generally claim that a theoretical foundation contributes to effectiveness [6, 12–16], although a direct link has not yet been established.

In health promotion research, a large number of theories coexist that aim to understand health-related behaviour and provide tools for behaviour change. The Social Learning/Cognitive Theory (SCT), Theory of Reasoned Action/Planned Behaviour (TRA/TPB), and Health Belief Model (HBM) are the most dominant theories, more recently joined by the Stages of Change (SoC) and Social Ecological Model (SEM) [17–21].

The SCT posits that people acquire and maintain particular behavioural patterns through a constant interaction between three factors: environment, personal factors, and behaviour [22, 23]. Behaviour is not simply the result of the environment and the person, just as the environment is not merely a function of the person and behaviour [17]. The HBM is based on an understanding that a person will take a health-related action if that person believes s/he is susceptible to the condition (perceived susceptibility), that the condition has serious consequences (perceived severity), that taking action would reduce their susceptibility to the condition or its severity (perceived benefits), and that these benefits outweigh the cost of taking action (perceived barriers). Action is taken more easily if the person is exposed to factors that prompt action (cues to action) and is confident in her/his ability to successfully perform an action (self-efficacy) [20, 24–26]. By contrast, the TRA suggests that a person's behaviour is determined by her/his intention to perform the behaviour. This intention is predicated by their attitude toward the specific behaviour and by beliefs about whether individuals who are important to the person approve or disapprove of the behaviour (subjective norm). The TPB includes an additional determinant: the belief s/he has control over a particular behaviour (perceived behavioural control) [20, 27, 28]. SoC theory argues that, in order to change a behaviour, an individual passes through five stages: precontemplation, contemplation, preparation, action, and maintenance [29]. People at different stages have different informational needs and benefit from interventions tailored to their particular stage [20]. The SEM identifies a number of interacting levels that influence behavior (individual, interpersonal, organizational, community, and public policy). According to this model, behaviours are shaped by the social environment [20, 30].

These dominant theories work at various levels and for different purposes. While the HBM and TRA/TPB are explanatory theories operating at the individual level, the SCT and SEM include the interpersonal and environmental levels, respectively. The SoC theory, in turn, is a change theory, not explaining a particular behaviour, but providing a framework for how people alter their behaviour.

With the overarching objective of improving effectiveness of HIV prevention interventions that target young people's sexual behaviour in sub-Saharan Africa, this paper examines the extent to which these interventions are grounded in theory, how these theories are applied and assesses if theory-based interventions are more effective in modifying sexual behaviour than interventions not explicitly grounded in theory.

## 2. Methods

### 2.1. Study Eligibility, Literature Search, and Data Extraction

We performed a systematic review to locate evaluated interventions that aim to reduce sexual risk behaviour of young people in sub-Saharan Africa. Studies were considered eligible if they reported on the evaluation of an HIV prevention intervention for young people on the subcontinent, had a control group, and were published between January 1990 and March 2012. Further, to be included, studies had to report on the general population of young people (10–25 years) and the intervention needed to aim to prevent HIV transmission by reducing sexual risk taking. Searches were performed in the online databases Medline (PubMed interface), ISI Web of Science, and EBSCOhost. Additional articles were identified on websites of international organisations and through searching references of eligible articles. Data extraction was then done in duplicate by five investigators using a predesigned and pretested extraction sheet. Further details of the search terms, study eligibility, and data extraction are detailed elsewhere [4].

### 2.2. Study Measures

We extracted data on characteristics of the interventions and theory use. Firstly, whether any theory had been used and, if so, which. Secondly, for what purpose the theory was used. We extracted full-text descriptions of how the theory had been used, which was later recoded into three categories: theory used to inform the intervention (e.g., for curriculum development); theory used to guide evaluation (e.g., to develop indicators); or both. Thirdly, a binary variable was derived, capturing whether an explanation was provided about why this theory was chosen. For the studies not reporting the use of a theory, we looked at the topics dealt with in the interventions and the envisaged interventions' outcomes. This gives us an indication of the underlying theoretical assumptions used in these interventions.

Data were also extracted on the behavioural outcomes of the interventions: condom use (at last sex; consistency and intention), sexual behaviour (primary abstinence; the proportion of sexually active youth; recent sexual intercourse; number of sexual partners and multiple partnerships), and biological outcomes (HIV/STI incidence). 

## 3. Results

1073 article titles and/or abstract were screened. After analysis of title and abstract, we reviewed 73 full-text publications. In total, evaluations of 34 studies met the inclusion criteria, reported on in 38 articles.  Table 1 sums the main intervention characteristics and study designs.

### 3.1. Theoretical Basis of the Interventions

About three quarters of the studies—25 of 34—mentioned having used at least one theory. In total, 19 different theories were mentioned 42 times. Several stated that they had applied two or more theories, with three papers reporting that the intervention design drew on three theories.

Of all the theories mentioned, the SCT was most prominent (*n* = 13). Other theories that were mentioned more than once are the HBM (*n* = 7) and the TRA/TPB (*n* = 6). Four studies mentioned using a behaviour change framework: behaviour changes for interventions model [37], applied behaviour change framework [44], steps to behaviour change framework [48] and stage theory of behaviour change [51]. Assessment of the concepts used in the interventions not explicitly mentioning the use of a theory indicated that they also operated from an assumption that knowledge, attitudes, beliefs, and/or role models determine sexual behaviour. Hence, it seems that most interventions are implicitly or explicitly guided by cognitive behavioural frameworks. The one exception is Baird (2012); this intervention uses an indirect pathway to try to influence HIV incidence, namely, through encouraging girls' school attendance.

Description of the main activities indicates that most interventions use one or a combination of participatory learning techniques, such as drama plays, poetry, songs, club formation, peer education (role modelling), and discussions and debates. This suggests that the learning strategies of most interventions were based on participatory learning approaches.

A small, but considerable proportion of interventions [32, 34–36, 38–40, 58, 62, 64] go beyond focusing on the individual young person and facilitate community involvement in the interventions. Here, the implicit theoretical assumption is that in order to change the participants' sexual behaviour, the community needs to be involved (cf. SEM).

There is no clear evolution detectable over time in the frequency of use of different theories; of the 20 studies which began in the decade 1990–1999, 14 reported theory use, while 11 of the 14 beginning after 2000 used theory.

### 3.2. Use of Theory in the Research Projects

Of 25 interventions that mentioned a theory, 7 said that the theory was used to both inform the content of the intervention (e.g., the curriculum) and to inform the evaluation or questionnaire design. In 13 studies, theory was reportedly used only to inform the intervention content, and in 4 only for designing the evaluation or questionnaire. One study mentioning theory use did not specify how this was applied [36]. The SCT was almost exclusively used to inform the intervention. The HBM was mostly used for evaluation purposes, predominately in studies from Population Services International [31, 33, 45, 55, 67], as was the TRA/TPB.

Only three articles provided information on why a particular theory was selected [41, 56, 60]. Nine authors limited themselves to a brief explanation of the theory itself [31, 37, 42, 45, 48, 50, 51, 63, 67]. The remainder did not provide any information on theory selection.

### 3.3. Theory Use and Intervention Effectiveness

Overall, the behavioural outcomes of the 34 studies were markedly heterogeneous, with little reduction in heterogeneity after stratifying by theory use (Table 2). It was not possible to discern any patterns in differential effectiveness between the role of theory in a study, or between studies reporting or not reporting theory use. Nor did we find particular differences in intervention design by theory use.

Four studies reported biological measures of intervention effectiveness [39, 40, 58, 59, 62]. Jewkes succeeded in reducing HSV-2 incidence. Baird's study, not explicitly based on theory, reported a reduced HIV incidence and HSV-2 incidence in the intervention group as compared to the control group, but these data were not controlled for baseline prevalence and should be treated with some caution [62]. Since the three other studies reporting biological measures all based their intervention on a theory, it is not possible to compare the effectiveness of theory- and non-theory-based interventions in changing these outcomes.

### 3.4. Evaluation of the Theory

Four studies refer to their theoretical basis in their conclusions, criticizing the theory, specifically “the theoretical approaches underlying the program have built in shortcomings which could result in the program not having significant impact on the students' behavioural intentions” [69]; “the discrepancies in the findings may be substantiated by the lack of systematic information that was available on the empirical and theoretical underpinnings upon which the KwaZulu-Natal Department of Education's program was based—a finding similar to *reports* of those educational programs that were not grounded in a theoretical understanding of adolescent sexual behaviour […]” [54]; “These findings present mixed evidence regarding the relationship between self-efficacy and outcome expectations and HIV protective behaviours among adolescents in Swaziland.” [63]; “TPB has received considerably more support from research for its predictive power of safe sex behaviour than the HBM.” [42].

## 4. Discussion

The review found that the majority of HIV prevention interventions targeted at youth in sub-Saharan Africa use theory-based approaches. A wide range of theories have been employed, but three behavioural theories predominate: SCT, HBM, and TRA/TPB. No one theory emerged dominant, as reporting on the choice, use, and specific evaluation of theory was low.

### 4.1. Comparison with Other Reviews

Broadly, the results are consistent with reviews of HIV risk-reduction interventions elsewhere, though some variation in use of theory can be noted across these reviews. Pedlow and Carey [70] reviewed 23 randomized controlled trials of HIV risk-reduction interventions for adolescents in the United States and found an explicit theoretical rationale in all but one study. Similar to our review, SCT was most common (18/23). Three other theories were used in four or more studies (TRA, HBM, and Information-Motivation-Behavioural Skills Model). A review on the impact of HIV and sex education programs on youth throughout the world [6] found that more than four fifths of the 83 interventions identified one or more theory. SCT formed the basis for more than half (54%) of these interventions. TRA (19%), HBM (12%), TPB (10%), and the Information-Motivation-Behavioural Skills Model (10%) were also commonly mentioned. Two other reviews covering HIV, STD, or pregnancy risk-reduction interventions among adolescents in the United States had comparable results, with a similar distribution of theories used [12, 71]. While several new theories or integrated models have been developed since the outbreak of HIV focussing specifically on sexual health behaviours like condom use [8, 72], they are not used in HIV interventions for young people in sub-Saharan Africa.

### 4.2. Gaps in (the Use of) Theory

By focussing on cognitive constructs of behaviour, the interventions explicitly or implicitly start from the assumption that cognitions influence the person's thinking and decision making, and thus drive sexual behaviour [73]. In the remaining discussion, we will focus on the utility of grounding HIV prevention interventions for young people on a cognitive behavioural framework. We attempt to identify critical areas for attention and improvement on different levels.

Firstly, cognitive behavioural models aim to explain a particular behaviour. The theoretical constructs that influence behavioural decisions may vary, depending on the behaviour in question. This poses marked challenges for HIV prevention interventions, since they generally attempt to influence a wide range of behaviours—for example, increasing condoms use, reducing the number of sexual partners, minimising sexual activity, delaying the onset of sexual debut—which are influenced by different factors. To further complicate matters, sexual decisions may vary depending on the reasons for sexual intercourse (ranging between, e.g., intimacy or desire, external factors, and affect management). These, in turn, are further influenced by gender and psychological characteristics (e.g., depression, self-esteem, and impulsiveness) [74]. Thus, sexual behaviour itself is far from a uniform behaviour, but rather a collection of several relatively distinct behaviours, that can be shaped by different factors in different contexts. While the use of a theoretical framework provides grip in structuring an HIV prevention intervention, the interventionist needs to be very clear about what behaviour they aim to alter and which factors determine this behaviour.

Second, the applicability of cognitive behavioural models to youth sexual behaviours may vary between development stages. For instance, applying cognitive theory to young people with no or limited sexual experience may be difficult. This group may not yet have well-anchored ideas, and consequently their attitudes, norms, and beliefs about safe sexual behaviour may be less clear and stable than for their adult counterparts [70, 73]. Theories used in HIV interventions targeted at youth could be strengthened by accounting for the extent to which individual decision making is supported by one's age, gender, or other personal characteristics.

Third, these theories seem to ignore the fact that sexual intercourse takes place between two persons, within a relationship. Sexual decisions do not depend on the individual, but also on the sexual partner and the type of relationship. Young people might have specific types of partners that may influence sexual decision making. For example, relationships with someone who is much older are risky because it exposes the younger person (mostly girls) to a partner who is more likely to be sexually experienced and hence more likely to be HIV-positive [75, 76]. Often, these age-disparate relationships are transactional in nature, with money or gifts given in exchange for sexual intercourse [75, 77–81]. Also, young people in same-age relationships might have different types of relationships than adults. They tend to be in what is called by Bastard et al. [82] the “courtship-seduction” phases of relationships, in which the predominant concerns are to “present the best image, win trust, and avoid sources of conflict. These concerns take precedence over that of protecting oneself from the risk of AIDS.”

Fourth, while some interventions recognize the importance of involving the community, only the SCT explicitly stresses the influence of contextual and structural factors on an individual's behaviour. Even though the theory states that the social environment is an important determinant of the behaviour, many interventions based on SCT did not attempt to include or influence environmental factors. Most interventions are limited to providing information and teaching skills. TRA/TPB implicitly includes this level by stating that personal attitudes, and norms are influenced by behavioural and normative beliefs in the society, which is useful for tracking varying modes of sexual socialisation. However, this is an indirect effect of the environment on individual behaviour, while still ignoring the broader structural factors that shape sexual behaviour. Many recent studies have demonstrated the contribution of structural factors to young people's vulnerability for HIV [75, 83–85]. These environmental aspects include both distal influences—such as taboos on adolescent sexuality, norms and values, policies, poverty, education as well as more proximate influences. These include families' opinions about adolescent relationships or teachers refusing to talk about condoms. Increased efforts in future studies to account for structural factors at a theoretical level may improve the design of interventions and assist in their evaluation, by understanding the possible barriers between motivation and actual behaviour change.

According to Gielen and Sleet [86], behavioural interventions can be subdivided into three categories, those aimed at intrapersonal factors (e.g., knowledge, skills, and intentions); interpersonal factors (including relational motivations and social desirability); community factors (e.g., culture, gender inequalities, poverty, and violence). We have already argued that the most common theories used in HIV interventions directed to youth do not adequately address interpersonal factors, the failure to account for contextual factors further compounds the difficulty of evaluating interventions and understanding the possible barriers between motivation and actual behaviour change. 

Finally, while cognitive behavioural theories of change might be successful in altering cognitions and behavioural intentions, they provide insufficient directives on translating this into actual behaviour change. Thus interventions could be regarded as successful in having altered motivations and intentions, even though behavioural change may not result. Similarly, interventions based on the HBM, might increase the perceived severity and susceptibility of a person, and relieve barriers to behaviour change, but in itself, might be insufficient to alter the sexual behaviour. Clearly, motivations or beliefs about behaviour change on a cognitive or rational level need to be accompanied by a clear strategy for introducing a new behaviour [87].

## 5. Conclusion

In the end, it boils down to two key questions: what determines sexual behaviour of young people? And what frameworks are most useful for making sense of and impacting positively on determinants of youth sexual behaviour? Recognizing the complexity and heterogeneity of this particular behaviour, theory can provide help in generalizing key determinants and making them operational. Theories aim to describe determinants and processes that account for or guide behaviour (change) through the rationalization of individual decisions. This aids in understanding human behaviour, and when used appropriately, can provide a solid grounding for program development and evaluation. The strength of theory is to generalize and simplify complex situations. However, in the case of HIV prevention interventions for young people, the dominant theories might oversimplify sexual behaviour. While such cognitive behavioural models can explain the links between intention and behaviour, particularly at an intrapersonal level, they are less able to account for interpersonal and contextual factors related to the complexity of sex, the experience of youth and disparities in social, cultural, and economic realities of youth in sub-Saharan Africa.

## Figures and Tables

**Table 1:** Characteristics of studies included in systematic review of use of behavioural theory in HIV prevention interventions in youth in sub-Saharan Africa.

Author, year	Country	Year of intervention	Study design	Sample size at baseline (males/females)	Main intervention activities (duration)	Intervention setting (urban/rural)	Theory or theories used	Role of theory in the study	Explanation provided about why theory used?
Central Africa									

Van Rossem and Meekers [31]	Cameroon	1996-1997	Repeat C/S, quasiexperimental	1606 (753/757)	Behaviour change communication and promotion through peers and in media, condom distribution, youth-friendly services (13 months)	Community (urban)	Health Belief Model	Development of intervention and questionnaire/evaluation	No

Speizer et al. [32]	Cameroon	1997-1998	Repeat C/S, quasiexperimental	802 (400/402)	Through discussion groups, one-on-one meetings, and health and sport association gatherings, peer educators informed their peers and referred them to services. Promotional materials were distributed in schools and community (18 months)	School + Community (urban)	NR, focus on knowledge	NA	NA

Meekers et al. [33]	Cameroon	2000-2001	Repeat C/S, pre post-controlling for exposure	1956 (1056/900)	Media and interpersonal communication campaign. Peer education, magazine, radio drama, radio call-in show, media campaign, condom promotion (12 months)	Community (urban)	Health Belief Model, Social Learning Theory, Theory of Reasoned Action	Development of intervention and questionnaire/evaluation	No

Eastern Africa									

Klepp et al. [34, 35]	Tanzania	1990	Cohort, randomized schools	1063 (502/561)	Teachers provided information, students created posters and performed songs, poetry, drama and role-play, small-group discussions among students. Interviews and panel discussions with parents and community members (2-3 months)	Primary school (urban + rural)	Social Learning Theory and Theory of Reasoned Action	Development of intervention and questionnaire/evaluation	No

Shuey et al. [36]	Uganda	1994–1996	Repeat C/S, quasiexperimental	800 (398/402)	Strengthen existing school health curriculum, meeting with parents and community leaders, formation of school health clubs with peer education, question boxes (2 years)	Primary school (urban + rural)	Social Cognitive Theory	NR	No

Kinsman et al. [37]	Uganda	1997-1998	Cohort, quasiexperimental	2077 (920/1157)	Extracurricular classes by trained teachers (1 year)	Primary and secondary schools (rural)	Behaviour Changes for Interventions Model	Development of intervention and questionnaire/evaluation	No

Erulkar et al. [38]	Kenya	1998–2000	Repeat C/S, quasiexperimental	1544 (792/752)	Adult counsellor in community educating youth, referral to youth-friendly services and encouraging parent-child communication (3 years)	Community (urban + rural)	NR, focus on values, knowledge, gender, and empowerment	NA	NA

Ross et al. [39], Doyle et al. [40]	Tanzania	1998–2002	Repeat C/S, randomized communities	Ross: 9219 (5103/4116) Doyle: 13814 (7300/6514)	Participatory, teacher-led, peer-assisted, in-school program, youth-friendly health services, condom promotion and distribution, and youth health days and video shows in community (3 years)	School + Community (rural)	Social Learning Theory	Development of intervention	No

Maticka-Tyndale et al. [41]	Kenya	2002-2003	Repeat C/S, randomized schools	7392 (3636/3764)	Peer education on level of teachers and students, question boxes, school health clubs, information corners and assemblies, drama, music and literary performances (18 months)	Primary school (urban + rural)	Social Learning Theory and Scripting Theory	Development of intervention	Yes

Rijsdijk et al. [42]	Uganda	2008	Cohort, randomized schools	1986 (1096/889)	low-tech, computer-based, interactive comprehensive sex education programme, teacher-led (6 months)	Secondary school (urban + rural)	Theory of Planned Behavior and Health Belief Model	Development of intervention	No

Southern Africa									

Kuhn et al. [43]	South Africa	1990	Repeat C/S, quasiexperimental	567 (not reported)	Intense, high-profile focus on AIDS in the school by teachers (2 weeks)	Secondary school (urban)	NR, focus on knowledge and attitudes	NA	NA

Harvey et al. [44]	South Africa	1993-1994	Cohort, randomized schools	1080 (447/633)	“School open day” with drama, song, dance, poetry, and posters prepared and presented by students (3 days)	Secondary school (urban + rural)	Applied behaviour change framework	Development of questionnaire/evaluation	No

Meekers [45]	South Africa	1994–1997	Repeat C/S, quasiexperimental	226 (0/226)	Mass media campaign, peer education and condom promotion and distribution (35 months)	Community (urban)	Health Belief Model	Development of questionnaire/evaluation	No

Fitzgerald et al.; Stanton et al. [46, 47]	Namibia	1996	Cohort, randomized participants	515 (236/279)	Curriculum taught by a teacher and out-of-school youth (7 weeks)	Secondary school (urban + rural)	Social Cognitive Theory/Protective Motivational Theory	Development of intervention and questionnaire/evaluation	No

Kim et al. [48]	Zimbabwe	1997-1998	Repeat C/S, quasiexperimental	1426 (713/713)	Mass media campaign, community drama groups, peer educators, youth-friendly health services (6 months)	School + Community (urban)	Steps to Behaviour Change Framework	Development of intervention	No

James et al. [49]	South Africa	1998	Cohort, randomized schools	1168 (542/616)	Reading of a comic book (1 hour)	Secondary school (urban + rural)	Theory of Health Promotion and Social Learning	Development of intervention	No

Visser [50]	South Africa	1998–2000	Repeat C/S, pre post-controlling for exposure	873 (410/463)	Trained teachers and professionals provide life skills and HIV/AIDS education. Parents included in action committee (1 year)	Secondary school (urban)	Health Belief Model	Development of intervention	No

Underwood et al. [51]	Zambia	1999-2000	Repeat C/S, quasiexperimental	921 (378/543)	Participatory developed mass media campaign (7 months)	Community (urban + rural)	Stage Theory of Behaviour Change	Development of intervention	No

Magnani et al. [52]	South Africa	1999–2001	Cohort, pre post-controlling for exposure	3052 (1375/1677)	Life skills curriculum taught by teachers (2 years)	Secondary school (urban)	Social Learning Theory	Development of intervention	No

Agha [53]	Zambia	2000	Cohort, randomized schools	481 (268/213)	Peer educators using discussion and drama skits (1 hour 45 min)	Secondary school (urban)	NR, focus on knowledge, normative beliefs, and risk perception	NA	NA

James et al. [54]	South Africa	2001	Cohort, randomized schools	936 (456/466)	Life skills intervention taught by trained teachers (20 weeks)	Secondary school (urban + rural)	Social Cognitive Theory and Theory of Planned Behaviour	Development of questionnaire/evaluation	No

Plautz et al. [55]	Madagascar	2001-2002	Cohort, pre post-controlling for exposure	1785 (1000/785)	Youth-friendly services, mass media, and interpersonal communication by peer educators (23 months)	Community (urban + rural)	Social Learning Theory, Health Belief Model, and Theory of Reasoned Action	Development of intervention and questionnaire/evaluation	No

Karnell et al. [56]	South Africa	2002	Cohort, randomized schools	661 (324/337)	Peer educators using recorded monologues of fictional characters, teacher support (8 weeks)	Secondary school (urban)	Social Learning Theory, Social Inoculation, Cognitive Behaviour Theory	Development of intervention and questionnaire/evaluation	Yes

Visser [57]	South Africa	2002-2003	Repeat C/S, quasiexperimental	1918 (858/1060)	Peer education (18 months)	Secondary schools (urban)	Systems Theory	Development of intervention	No

Jewkes et al. [58, 59]	South Africa	2003-2004	Cohort, randomized communities	2776 (1360/1416)	Participatory learning approaches taught by facilitators, peer group meeting, community meeting (6–8 weeks)	Community (rural)	Participatory Learning Approach and Adult Education Theory	Development of intervention	No

Tibbits et al. [60]	South Africa	2004-2005	Cohort, randomized schools	4040 (2020/2020)	Comprehensive, risk-reduction life skills curriculum for adolescents, teacher-led (24 months)	Secondary school, urban	Selective optimization with compensation, Self-Determination Theory, and Social Cognitive Theory	Development of intervention	Yes

Mason-Jones et al. [61]	South Africa	2007-2008	Cohort, quasiexperimental	3934 (1661/2211)	Government-led peer education project, in class standard curriculum, conversations outside class, referral (18 months)	Secondary school (urban + rural)	NR, knowledge and psychosocial characteristics	NA	NA

Baird et al. [62]	Malawi	2008-2009	Cohort, randomized schools	3796 (0/3796)	Monthly cash transfer programme to reduce the risk of STI infection (24 months)	School + community (urban + rural)	NR, focus on structural factor (poverty and education) and knowledge	NA	NA

Burnett et al. [63]	Swaziland	NR	Cohort, randomized youth	204 (101/103)	Teacher-led life-skills HIV prevention education program, curriculum, interactive techniques, role playing, and group discussions (13 weeks)	Secondary school (urban)	Self-efficacy theory and Protection Motivation Theory	Development of intervention	No

Western Africa									

Brieger et al. [64]	Nigeria and Ghana	1994–1997	Repeat C/S, quasiexperimental	1784 (not reported)	Peer educators, promotion of community-level networks, referral to services (30 months)	School + Community (urban)	NR, focus on knowledge and attitudes	NA	NA

Fawole et al. [65]	Nigeria	1996	Cohort, pre post-controlling for exposure	450 (204/246)	Education sessions by community physicians with help of teachers (1 month)	Secondary school (urban)	NR, focus on knowledge and attitudes	NA	NA

Okonofua et al. [66]	Nigeria	1997-1998	Repeat C/S, randomized schools	1896 (877/1008)	Establishment of reproductive health club in school, health awareness campaigns by professionals, distribution of print material, peer education, youth-friendly services (11 months)	Secondary school (urban)	NR, focus on knowledge and barriers	NA	NA

Van Rossem and Meekers [67]	Guinea	1997-1998	Cohort, quasiexperimental	2016 (925/1091)	Peer educators (discussion and theatre), condom promotion, billboards, youth-friendly services and contraception distribution (8 months)	Community (urban)	Health Belief Model	Development of questionnaire/evaluation	No

Atwood et al. [68]	Liberia	2007-2008	Cohort, randomized schools	812 (455/357)	Curriculum-based program by health educators (8 weeks)	Primary school (urban)	Social Cognitive Theory and Theory of Reasoned Action	Development of intervention	No

C/S: Repeated cross-sectional design.

NR: No theory is explicitly reported, dominant constructs used in the intervention.

NA: Not applicable.

**Table 2:** Description of study outcomes stratified by role of theory use in each study.

	Condom use at last sex	Ever/consistently used condom	Sexual debut, proportion of sexually active youth	Sexual intercourse in past months	Number of sexual partners	HIV incidence	HSV-2	Other STIs	Overview
Theory used for development of intervention	Visser, 2005^°^	Ross, 2007^++^	Visser, 2005^−−^	Maticka-Tyndale, 2007^°^	Visser, 2005^°^	Ross, 2007^°^	Ross, 2007^°^	Ross, 2007^°^	44 outcomes
	Magnani, 2005^++^	Underwood, 2006^++^	Magnani, 2005^−−^	Visser, 2007^++^	Magnani, 2005^++^	Jewkes, 2008^°^	Jewkes, 2008^++^	Jewkes, 2008^°^	^ ++^7
	Maticka-Tyndale, 2007^+^	Kim, 2001^°^	Maticka-Tyndale, 2007^+^	Kim, 2001^+^	Ross, 2007^+^	Doyle, 2010^°^	Doyle, 2010^°^	Doyle, 2010^°^	^ +^10
	Ross, 2007^+^	Magnani, 2005^++^	Ross, 2007^°^		Visser, 2007^−−^				^ °^22
	Jewkes, 2008^°^	Atwood 2012^°^	Klepp, 1997^+^		Kim, 2001^+^				^ −^1
	Underwood, 2006^°^		Kim, 2001^+^		Atwood, 2012^−−^				^ −−^4
	Visser, 2007^°^		Underwood, 2006^−^		Doyle, 2010^+^				
	Doyle, 2010^°^		Atwood, 2012^+^						
	Tibbits, 2011^°^		Burnet, 2011^°^						
			Doyle, 2010^°^						
			Tibbits, 2011^°^						

	James, 2006^+^	Harvey, 2000^++^	Harvey, 2000^°^	James, 2006^+^	Harvey, 2000^°^				12 outcomes
	Meekers, 1998^°^	Meekers, 1998^°^	Van Rossem, 1999^+^		Van Rossem, 1999^°^				^ ++^1
Theory used for development of evaluation or questionnaire	Van Rossem, 1999^+^	Van Rossem, 1999^+^	Meekers, 1998^°^						^ +^5
									^ °^6
									^ −^0
									^ −−^0

	Van Rossem, 2000^°^	Van Rossem, 2000^++^	Fitzgerald, 1999^°^	Fitzgerald, 1999^°^	Fitzgerald, 1999^°^				15 outcomes
	Fitzgerald, 1999^°^	Meekers, 2005^+^	Van Rossem, 2000^°^		Van Rossem, 2000^+^				^ ++^1
Theory used for development of intervention and evaluation or questionnaire	Meekers, 2005^+^	Fitzgerald, 1999^°^	Plautz, 2003^−^						^ +^3
	Plautz, 2003^°^	Plautz, 2003^°^							^ °^10
	Karnell, 2005^°^								^ −^1
									^ −−^0

Theory used, but uncertain in which phase of study			Shuey^++^						1 outcome
									^ ++^1

	Speizer^+^	Agha, 2002^°^	Speizer^++^	Speizer^−−^	Agha, 2002^++^	Baird, 2012^++^	Baird, 2012^++^	Baird, 2012^°^	18 outcomes
	Agha, 2002^°^	Okonofua^++^	Brieger, 2001^−−^	Agha, 2002^++^	Fawole, 1999^°^				^ ++^6
Interventions not explicitly based on theory	Erulkar, 2004^+^	Kuhn^°^	Fawole, 1999^°^	Erulkar, 2004^°^	Erulkar, 2004^+^				^ +^4
	Fawole, 1999^°^	Fawole, 1999^°^	Erulkar, 2004^+^						^ °^12
	Mason-Jones, 2011^°^	Baird, 2012^°^	Baird, 2012^°^						^ −^0
			Mason-Jones, 2011^−−^						^ −−^3

^++^significant positive intervention effect on outcome variable for the whole study population.

^+^significant positive intervention impact on outcome variable for a subgroup of the target population, and no significant impact on the whole study population or whole population impact not reported.

^°^no significant intervention impact on the outcome variable.

^−^significant negative intervention effect on outcome variable in a sub-group of the target population, and no significant impact on the whole study population or whole population impact not reported.

^−−^significant negative intervention effect on the outcome variable for the whole study population.
